# Plastid Proteomic Analysis in Tomato Fruit Development

**DOI:** 10.1371/journal.pone.0137266

**Published:** 2015-09-15

**Authors:** Miho Suzuki, Sachiko Takahashi, Takanori Kondo, Hideo Dohra, Yumihiko Ito, Yoshikazu Kiriiwa, Marina Hayashi, Shiori Kamiya, Masaya Kato, Masayuki Fujiwara, Yoichiro Fukao, Megumi Kobayashi, Noriko Nagata, Reiko Motohashi

**Affiliations:** 1 Faculty of Agriculture, Shizuoka University, Shizuoka city, Shizuoka, Japan; 2 Instrumental Research Support Office, Research Institute of Green Science and Technology, Shizuoka University, Shizuoka city, Shizuoka, Japan; 3 The Plant Science Education Unit, Nara Institute of Science and Technology, Ikoma city, Nara, Japan; 4 Faculty of Science, Japan Woman’s University, Bunkyo-ku, Tokyo, Japan; Cairo University, EGYPT

## Abstract

To better understand the mechanism of plastid differentiation from chloroplast to chromoplast, we examined proteome and plastid changes over four distinct developmental stages of ‘Micro-Tom’ fruit. Additionally, to discover more about the relationship between fruit color and plastid differentiation, we also analyzed and compared ‘Micro-Tom’ results with those from two other varieties, ‘Black’ and ‘White Beauty’. We confirmed that proteins related to photosynthesis remain through the orange maturity stage of ‘Micro-Tom’, and also learned that thylakoids no longer exist at this stage. These results suggest that at a minimum there are changes in plastid morphology occurring before all related proteins change. We also compared ‘Micro-Tom’ fruits with ‘Black’ and ‘White Beauty’ using two-dimensional gel electrophoresis. We found a decrease of CHRC (plastid-lipid-associated protein) and HrBP1 (harpin binding protein-1) in the ‘Black’ and ‘White Beauty’ varieties. CHRC is involved in carotenoid accumulation and stabilization. HrBP1 in *Arabidopsis* has a sequence similar to proteins in the PAP/fibrillin family. These proteins have characteristics and functions similar to lipocalin, an example of which is the transport of hydrophobic molecules. We detected spots of TIL (temperature-induced lipocalin) in 2D-PAGE results, however the number of spots and their isoelectric points differed between ‘Micro-Tom’ and ‘Black’/‘White Beauty’. Lipocalin has various functions including those related to environmental stress response, apoptosis induction, membrane formation and fixation, regulation of immune response, cell growth, and metabolism adjustment. Lipocalin related proteins such as TIL and HrBP1 could be related to the accumulation of carotenoids, fruit color and the differentiation of chromoplast.

## Introduction

In the long history of proteomics research, analysis of plastids has mainly focused on chloroplast [1–4]. The identification of the genome sequence of *Arabidopsis thaliana* set the stage for the use of mass spectrometry protein identification in rice and other crops, contributing greatly to the understanding of chloroplast biosynthesis at molecular levels [5–7]. Furthermore, there have been reports in the past decade on the proteomic data of various types of plastids such as etioplast in rice [6], proplastid in tobacco bright yellow-2 [7], amyloplast in wheat and potato [8, 9], and chromoplast in bell pepper [10]. However as there is no suitable method for isolating these plastids, there are fewer research reports on their proteomics than those of chloroplast. In such cases results do not go beyond the isolation of various types of plastids and the simple identification of proteins. There are no reports regarding the relationship of said plastid proteins to plastid differentiation and development.

In isolating chloroplasts, density gradient centrifugation methods using Percoll or sucrose are often utilized. However, it was reported that when etioplasts were isolated using Percoll density gradient centrifugation, the isolated etioplast aggregated, preventing the efficient separation of organelles [6]. Also it has been reported that when using sucrose density gradient centrifugation, isolated plastids were accompanied by other organelles. As an alternative, Nycodenz density gradient centrifugation is superior due to its simplicity and its ability to isolate pure plastids without contamination by other organelles or crushed plastids [6].

In recent years there have been several proteome analyses which have utilized improved methods of plastid isolation; Studies on chromoplast in tomato [11, 12], *Citrus* (sweet orange) [13] and another regarding six different crop species (watermelon, tomato, carrot, cauliflower, papaya and pepper) [14], to name a few. The only known report of proteomic analysis of chloroplast differentiating to chromoplast in tomato is one that analyzed and compared the proteome data of tomato fruit of 3 maturity stages (Mature-green, Breaker, Red). In this study the number of thylakoid proteins decreased with fruit maturation, while proteins related to energy production increased [12]. Furthermore, in an attempt to learn more about regulation via post-translational modifications during fruit ripening, a phosphoproteomic analysis of chromoplast of *Citrus* has also been reported [15].

Until the results of this study, little has been known about how Lipocalins are related to fruit maturation and plastid differentiation. Lipocalins are a small protein family found over a wide array of animals, plants, and microorganisms [16, 17]. In plants, lipocalins are categorized by sequence into TIL and CHL (chloroplast lipocalins). Lipocalin-like-proteins (proteins with very similar sequence to lipocalins) are categorized into Violaxnthin de-epoxidase (VDE) and Zeaxanthin epoxidase (ZE) [17, 18]. Studies conducted using plant *TIL* and *CHL* have mostly centered around *AtTIL* (*Arabidopsis thaliana TIL*) and *TaTIL* (*Triticum aestivum TIL*). *AtTIL* and *TaTIL* express under conditions of cold/heat stress [19]. While only 1 copy of *TIL* is found in the *Arabidopsis thaliana* genome, *TIL* homologs *TIL1* and *TIL2* have been found in monocots such as rice, wheat and barely [18].

Tomato (*Solanum lycopersicum*) is known as a climacteric fruit whose maturation is induced by ethylene [20]. Tomato is a vegetable crop valued commercially all over the world, and for this reason it is a model plant for fruit biology research in areas such as development, maturation and metabolism [21]. The genome sequence of tomato was successfully determined in 2012 [22, 23]. The chloroplast genome sequence of tomato was determined in 2006 [24]; a full length cDNA library was built and a MiBASE database (http://www.kazusa.or.jp/jsol/microtom/) was made by the Kazusa DNA Research Institute (Japan) [25]. These resources are invaluable and have significantly improved the status of *Solanaceae* research. Recently there have been many studies concerning tomato fruit ripening and development mechanisms, as well as transcriptome and metabolome analysis [26–29].

Carotenoids are synthesized in chromoplast in fruits, flowers and leaves. Tomato contains some carotenoids including a high amount of lycopene and beta-carotene, and the typical red color of tomato fruit is caused by the accumulation of all-trans-lycopene [30]. In the case of flavonoids, yellow tomato peels accumulate narigenin chalcone which is the first intermediate compound in flavonoid biosynthesis. It is known that this accumulation makes the dry weight of a cuticle increase by 1% in tomato fruit [31]. Iijima et al. identified 70 flavonoids and flavonoid derivatives in pericarps and peels of fruits at various developmental stages in ‘Micro-Tom’ [32].

‘Micro-Tom’ is an ideal material for studying differentiation from chloroplast to chromoplast also due to its relatively short ripening cycle (only 10~14 days to ripened red), and because peel color is linked closely to plastid change. In this study, proteomics techniques were used to identify chromoplast proteins of the ‘White Beauty’ and ‘Black’ varieties, as well as that of ‘Micro-Tom’ fruits in four developmental stages (mature green, yellow, orange and red) to better understand the relationships of fruit color, chromoplast development and plastid proteins. From the proteins identified, those found to be specific to the differentiation mechanism from chloroplast to chromoplast were further examined and analyzed.

## Materials and Methods

### Plant Material

**Fig 1:**
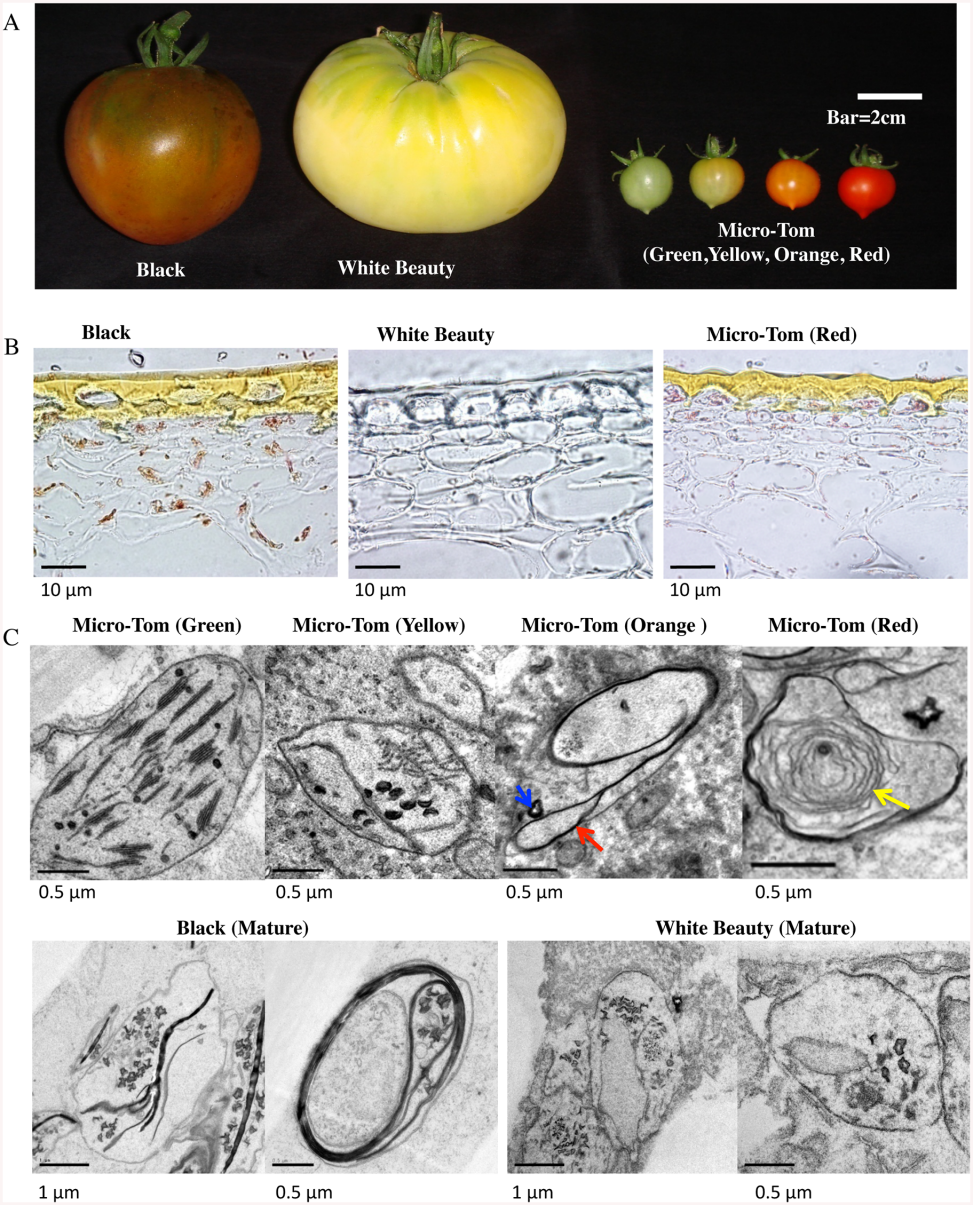
Tomato fruit material. A. Tomato Phenotypes. From left to right: ‘Black’, ‘White Beauty’, ‘Micro-Tom’ (from green stage to red stage) ‘Black’ and ‘White Beauty’ fruit fully ripens. For the 4 stages of ‘Micro-Tom’ shown, DPA (Day Post Anthesis) is 30~33 (green), 32~33 (yellow), 33~35 (orange), and 41~45 (red). B. Frozen sections of cuticle layers. From left to right: ‘Black’ mature stage, ‘White Beauty’ mature stage, ‘Micro-Tom’ (red maturity stage). The cuticle layers are found in the upper portion of each image. C. Electron microscopy of plastids in each tomato. Upper row shows Electron micrographs of plastids in each developmental stage of ‘Micro-Tom’. Lower row shows Electron micrographs of plastids in ‘Black’ and ‘White Beauty’ at mature stage. The bar in each figure represents the size scale for that figure. Red arrow shows membrane structures with high molecule density. Blue arrow shows crystal structure. Yellow arrow shows a whirled membrane structure.

‘Micro-Tom’ (*Solanum lycopersicum* cv. ‘Micro-Tom’, a model plant in the *Solanaceae* family), ‘White Beauty’ (Heirloom Tomato), and ‘Black’ (Heirloom Tomato) were used (Fig 1A).

‘Micro-Tom’ is a dwarf phenotype cultivar, originally first reported of in 1989, and was fixed by crossbreeding Florida Basket and Ohio 4013–3 (12th filial generation) [33]. ‘Micro-Tom’ seeds were provided by Prof. Ezura and Prof. Mizoguchi of Tsukuba University. ‘Micro-Tom’ was chosen as a model for many reasons—but mainly because of its size, ease of cultivation, and the availability of a full length cDNA clone (Aoki et al., KAZUSA DNA Institute) [25].

‘Black’ is an Heirloom cultivar that originated in Russia, having near-black (very dark red) fruit and black shoulder, and a slightly narrow fruit shape. ‘Black’ seeds and other tomato cultivar seeds except for ‘Micro-Tom’ were bought from Tomato Growers Supply Company (http://www.tomatogrowers.com). ‘White Beauty’ is a classic Heirloom cultivar that was first introduced in Michigan in 1927, with cream / white-colored fruit that are slightly flat and hourglass shaped. An allelism test on 6 types of white tomato was performed: (large fruit types) ‘White Beauty’, ‘White Queen’ and ‘Great White’; and (smaller fruit types) ‘Snow White’, ‘Dr. Carolyn’ and ‘Ghost Cherry’. No differences were found in their respective phenotypes, and it was determined that all 6 varieties originated from the same gene-disruption. This result, coupled with the fact that it is more resistant to disease than the other varieties, lead us to choose ‘White Beauty’ as the white tomato in our research. Material used to generate Fig 1 was taken from the area of the fruit between the middle and shoulder areas. Fruit fully ripens 8 weeks post anthesis (winter), and 7 weeks post anthesis (the beginning of summer). For the 4 stages shown, DPA (Day Post Anthesis) is 30~33 (green), 32~33 (yellow), 33~35 (orange), and 41~45 (red). ‘White Beauty’ and ‘Black’ were grown to maturity in a similar timeframe but w/o constant temperature conditions. It warrants mention here that for ‘Black’ material only the upper-half of the fruit was used (the position with green colored areas). Materials were grown hydroponically using the Enshi formula (KNO_3_ 808 mg/l, MgSO_4_⋅7H_2_O 492 mg/l, Ca(NO_3_)⋅4H_2_O 944 mg/l, NH_4_H_2_PO_4_ 152 mg/l, Otsuka house 5 (Otsuka AgriTechno. Co., Ltd) 50 mg/l) half diluted.

### Microscopy

For light microscopy, the pieces (about 5 mm^3^) were fixed with 2% glutaraldehyde and 4% paraformaldehyde in a 20 mM sodium cacodylate buffer, pH 7.0, at 4°C overnight. Fixed samples were embedded in Super Cryoembedding Medium (Leica Microsystems K.K., Tokyo, Japan) within liquid N_2_. Thin cryosections (25 μm thick) were cut on a JUNG CM 3000 cryostat (Leica, Wien, Austria), and transferred to glass slides. They were investigated with an Olympus BX51 microscope with a DP71 CCD camera (Olympus, Tokyo, Japan).

For transmission electron microscopy, the pieces (about 0.5 mm^3^) were fixed in 2% glutaraldehyde and 4% paraformaldehyde, which was buffered with 20 mM sodium cacodylate at pH 7.0 for 20 h at 4°C, and washed with the same buffer for 4 h at 4°C. Subsequently they were post-fixed with 1% KMnO_4_ in 50 mM cacodylate buffer for 2 h at 4°C. The fixed samples were run through an alcohol series and embedded in Spurr's resin (Polysciences Inc., PA, USA). Ultra-thin sections (70 nm thick) were cut with a diamond knife on an ULTRACUT E ultra-microtome (Leica, Vienna, Austria), and transferred to Formvar-coated grids. They were double-stained with 1% uranyl acetate for 20 min and with lead citrate solution for 15 min. After washing with distilled water, the samples were investigated using a JEM-1200 EX transmission electron microscope (Jeol, Tokyo, Japan).

### Sample Preparation for Protein Extraction

**Fig 2:**
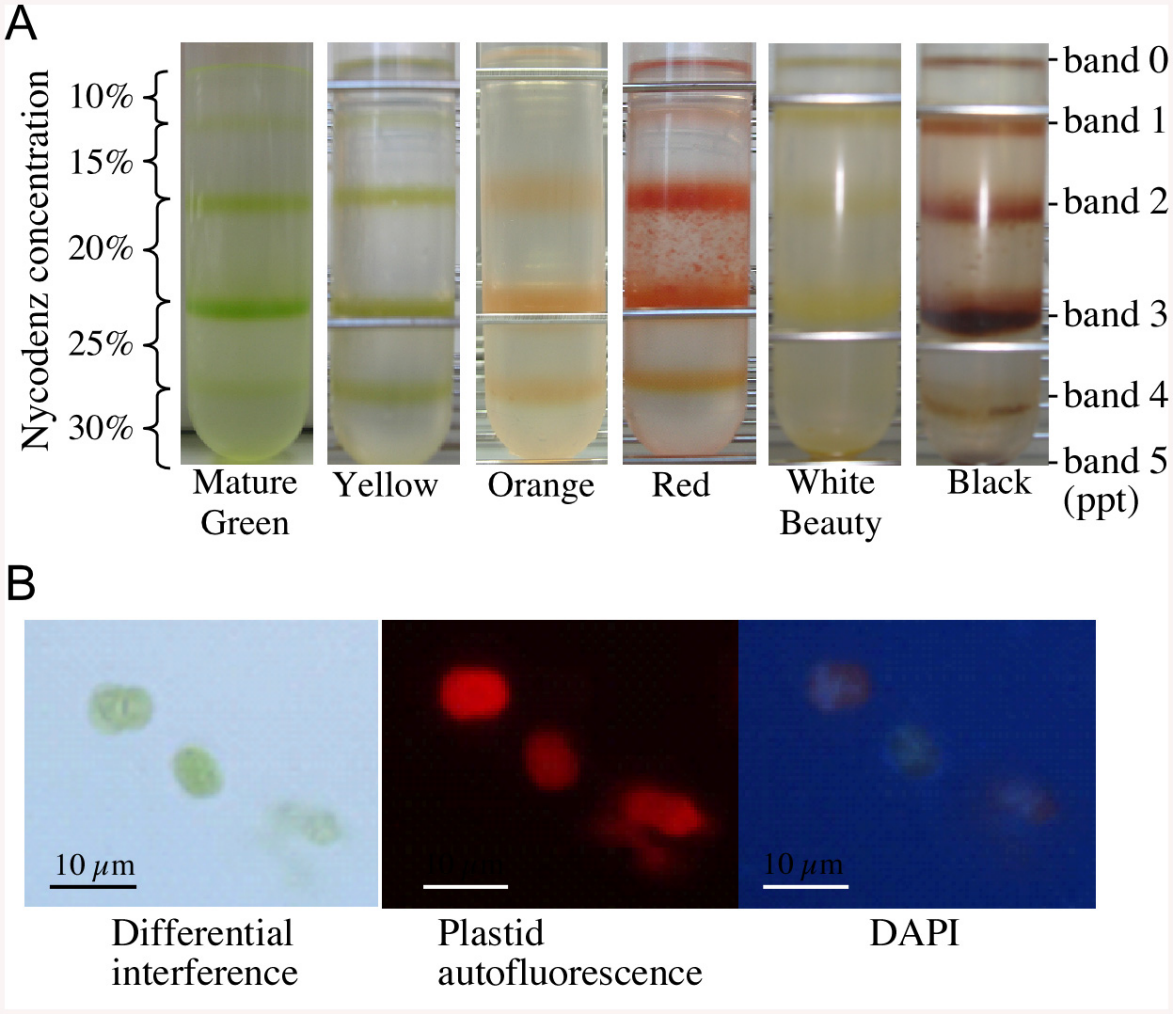
Isolation of plastids from tomato fruits. A. Plastids isolated from varying stages/varieties of tomato fruits using Nycodenz density gradient centrifugation. Left side (Y-axis) indicates Nycodenz concentrations. Bands 2 and 3 are plastid fractions. B. Observation of plastids by fluorescence microscopy. In order to determine whether or not the isolated plastids were intact, we employed fluorescence microscopy (Olympus SZX9), observing plastids by differential interference, autofluorescence at 572nm, and also by detecting fluorescence using DAPI (4’, 6-diamidiano-2-phenylindole) stain. The bar in each figure represents the size scale for that figure.

In previous research plastid isolation was attempted using Percoll, but there were many unwanted cell organelles, and isolated plastids were not intact. To solve this problem an etioplast isolation method was adopted, replacing Percoll with Nycodenz (Axis-Shield PoC AS, Oslo, Norway) [6]. About 100 g of each stage (30~33 DPA (green/mature green), 32~33 DPA (yellow), 33~35 DPA (orange), and 41~45 DPA (red)) of ‘Micro-Tom’, ‘Black’ and ‘White Beauty’ fruits (7 or 8 weeks DPA) were cooled in ice and homogenized in 300 ml of E.I.S. solution (10 mM HEPES/KOH pH 7.8, 2 mM EDTA, 2 mM MgCl_2_, 1 mM Tetrasodium pyrophosphate, 600 mM Sorbitol, 0.2% BSA (Albumin, from Bovine Serum, Cohn Fraction V, pH 7.0)), and after initial filtering with a tea strainer, filtered twice using a 4-layer Miracloth. Stepwise layering was performed using multilayered Nycodenz (50% Nycodenz: 10 mM HEPES/KOH pH 7.6, 2 mM EDTA, 2 mM MgCl_2_, 1 mM Tetrasodium pyrophosphate, 5 mM DTT (dithiothreitol)) at 25%, 20%, 15% and 10% concentrations. Disrupted cells of tomato were then layered from the 10% Nycodenz solution. Using a swing rotor (P28S; HITACHI and CP 100MX; HITACHI) the solution was centrifuged at 8,000g for 1 h. Band2 and band3 in the gradients contained the highest amount of plastids (Fig 2A). A comparison of the 2D-gel spot patterns of band2 and band3 found them to be identical, so the proteins were combined. As a result a total of 270 μg of plastid proteins were isolated from each of the 100 g fruit samples (‘Micro-Tom’, ‘Black’, ‘White Beauty’).

To confirm that we were isolating intact plastids, we observed them via microscope (Fig 2B). Minimal contamination by other organelle was confirmed based on an assay of marker enzymes of cytochrome c oxidase (COX; mitochondrial enzyme), NADPH-cytochrome c reductase (endoplasmic reticulum enzyme) and Inosine diphosphatase (IDPase; Golgi enzyme).

COX activity: 3.3 mg of cytochrome c (horse heart) was dissolved into 5 ml of 100 mM potassium phosphate buffer (pH 7.0). Base substrate was completed by the addition of 3.5 mg Na_2_S_2_O_4_ and reduced cytochrome c via airpump. 100 μl of isolated plastids were then added into 900 μl of substrate and inverted. The decrease of reduced cytochrome c was monitored by the decrease of OD_550_ at 30°C [34]. NADPH-cytochrome c reductase activity: after preincubating 900 μl of substrate solution (0.5 mg/ml cytochrome c, 50mM potassium phosphate buffer (pH 8.0), 1mM KCM (potassium cyanide), 0.14 mM NADPH, 1 μM antimycin A) at 30°C, 100 μl of isolated plastids were added and chemical reaction was initiated. The reduction of cytochrome c was monitored by the increase of OD_550_ at 30°C [35]. IDPase activity: after incubating 450 μl of substrate solution (5.3 mM IDP, 61 mM Tris (pH 7.5), 1 mM MgCl_2_) at 37°C for 5 min, 50 μl of isolated plastids were added and chemical reaction was initiated. After incubation for 1 h at 37°C, 50 μl were extracted and mixed with 750 μl phospha-C test kit (Wako). The mixed solution was incubated for 20 min at 37°C and chilled in ice water. Colorimetry was monitored by OD_750_ [36]. COX was detected a little in plastid solution (Where the COX activity in a solution combining band0 to band5 is 100%, the COX activity in the plastid solution was approximately 5%).

NADPH-cytochrome c reductase and IDPase were not detected in plastid solution.

### GeLC-MS/MS Analysis using LTQ-Orbitrap

Proteins were extracted in buffer (40 mM Tris-HCl (pH 6.8), 40 mM DTT, 5% SDS, protease inhibitor (Roche)), and insoluble materials were removed by centrifugation (30 min at 15,000g). Approximately 100 μg of protein per lane was loaded onto a 12.5% SDS gel (10 cm X 11 cm), and after electrophoretic separation of the proteins, the gel was stained with Coomassie blue. The gel lanes were cut into 7 pieces each and digested in Trypsin according to protocol in [37]. The resulting peptide samples were then examined using LC-MS/MS.

Trypsin-digested peptides were loaded onto a C18 column (100 μm internal diameter, 15 cm; L-Column, CERI) by HTC-PAL Autosampler (CTC Analytics). Two buffers (A and B) were used. Buffer A 0.1% (v/v) acetic acid and 2% (v/v) acetonitrile in water; Buffer B 0.1% (v/v) acetic acid and 90% (v/v) acetonitrile in water. Peptides were eluted from the column using a 25 min linear gradient of increasing Buffer B concentration (5% to 45%) applied by a Paradigm MS4 HPLC pump (Michrom BioResources). They were then introduced directly into an LTQ-Orbitrap mass spectrometer with a flow rate of 500 nl/min and a spray voltage of 2.0 kV. The set of MS scan range was m/z 450–1500 and the top three peaks were subjected to MS/MS analysis. The mass resolution for acquisition of precursor ion spectra was 30,000. The data of MS and MS/MS spectrum obtained from the LTQ-Orbitrap XL (Thermo Fisher Scientific) was compared against a protein database (taxonomy: Viridiplantae) from the National Center for Biotechnology Information (NCBI) using MASCOT Server software (version 2.1). The MASCOT MS/MS Ions Search (http://www.matrixscience.com/) [38] parameters were as follows: threshold offset to 0.05 in the ion-score cut off, peptide tolerance at 10 ppm, MS/MS tolerance at ±0.8Da, peptide charge of 2+ or 3+, Trypsin as enzyme allowing up to 1 missed cleavage, carbamidomethylation on cysteines as a fixed modification, and oxidation on methionine as a variable modification. Peptide data from GeLCMS that had a MASCOT search score of less than 30 was omitted. Comparing amino acid sequences obtained from our study with those in the Viridiplantae subset of the NCBInr database, matches with various plant types were found (such as rice, *Arabidopsis*, tobacco, and potato).

MASCOT DAT files were validated by Scaffold (Proteome Software, Inc.) (http://www.proteomesoftware.com/products/scaffold/) to increase confidence of the MASCOT search results. "Min Protein" which filters the results by Scaffold's probability that the protein identification is correct was set to 95.0%. "Min # Peptides" which filters the results by the number of unique peptides on which the protein identification is based was set to 1. "Min Peptide" which filters the results by requiring a minimum identification probability of a peptide from at least one spectrum was set to 95% or better.

Using the amino acid sequence of identified proteins, a BLAST search on TAIR (The Arabidopsis Information Resource) (http://www.arabidopsis.org/index.jsp) was conducted [39] searching for homologous *Arabidopsis* proteins (with an e-value less than 1e-10). The corresponding tomato proteins were annotated using PRO (Protein Ontology). Redundant proteins that were annotated to be the same *Arabidopsis* genes were removed. The dataset of non-redundant proteins was imported into the MapMan program (http://mapman.gabipd.org/web/guest/mapman) for functional classification [40]. Protein ontology enrichment analysis was performed on the BinGO website (http://www.psb.ugent.be/cbd/papers/BiNGO/Home.html) [41].

### Proteome analysis using 2D-PAGE (two-dimensional electrophoresis)

In order to acquire proteins from isolated plastids of fruits at 41~45 DPA (‘Micro-Tom’ Red stage) and 7 or 8 weeks DPA (‘White Beauty’ and ‘Black’) they were solubilized using a buffer (40 mM Tris-base, 7 M Urea, 2 M Thiourea, 0.5% Brij56, 2% CHAPS, protease inhibitor (Roche)). After 5~10 min of sonication, the solution was centrifuged at 15,000g/20°C for 30 min. Soluble proteins of the supernatants (also containing membrane proteins) were collected.

2D-PAGE was performed using GE Healthcare reagents and equipment. First dimension isoelectric focusing (IEF) was performed using an IPGphor3 (GE Healthcare) on 11 cm IPG DryStrips (Immobiline^TM^ DryStrip pH4-7; GE Healthcare). 100 μg of solubilized proteins were separated using the following electrophoresis conditions; at 20°C, 10 h at 100 μA, 1 min at 0-300V, 2 h at 300V, 1 h at 300~600V, 1 h at 600~1000V, 1 h at 1000~4000V, 12 h at 4000V.

Second dimension gel electrophoresis was performed using a SE600 Ruby (GE Healthcare) with 12% poly-Acrylamide gel (16 cm x 16 cm). Following electrophoresis, the gel was stained with Flamingo Fluorescent Gel Stain (BIO-RAD), and scanned using a Typhoon 9000 series (GE Healthcare). Using Image Master 2D Platinum 7.0 (GE Healthcare) a comparative analysis of protein spots was performed. Finally, using an Ettan Spot Picker (GE Healthcare) protein spots of interest in the gel were sampled. Isolated plastid protein members were reproduced more than three times using 2D-PAGE (data not shown).

LC–MS/MS analysis was performed by a linear ion trap time-of-flight mass spectrometer (LIT–TOF MS), NanoFrontier eLD (Hitachi High-Technologies Corporation) coupled to a nanoflow HPLC, NanoFrontier nLC (Hitachi High-Technologies Corporation). Peptides extracted from the gel were trapped and desalted with a C18 monolith trap column (0.05 mm ID×150 mm long; Hitachi High-Technologies Corporation) and then loaded onto a MonoCap C18 Fast-flow column (0.05 mm ID×150 mm long; GL Sciences, Inc., Tokyo, Japan) and eluted with a linear gradient from 2% to 40% solvent B in 60 min at a flow rate of 200 nl/min. Solvent A was 2% ACN and 0.1% formic acid, and solvent B was 98% ACN and 0.1% formic acid. The eluent was ionized with a nanoelectrospray ionization source equipped with an uncoated SilicaTip (New Objective, Woburn, MA, USA) and analyzed with a LIT–TOF MS. Mass spectra were obtained in positive ion mode at scan-mass range m/z 200–2000. MS/MS spectra were generated by collision-induced dissociation in the linear ion trap.

To identify the proteins in the gel pieces, MS and MS/MS data was converted to an MGF file using NanoFrontier eLD Data Processing Software (Hitachi High-Technologies Corporation) and analyzed with MASCOT MS/MS Ions Search using the following parameters: database, NCBInr; enzyme, trypsin; missed cleavages, 3; taxonomy, all entries; fixed modifications, carbamidomethyl (C); variable modifications, oxidation (HW) and oxidation (M); peptide tolerance, 0.2 Da; MS/MS tolerance, 0.2 Da; Peptide charge, 1+, 2+, and 3+; Instrument, ESI-TRAP. Results with MASCOT scores of less than 30 were omitted (similar to that of GeLCMS).

Using their respective GI numbers, information was obtained regarding the amino acid sequences of predicted proteins from the NCBI (National Center for Biotechnology Information) protein database (http://www.ncbi.nlm.nih.gov/), and subcellular protein localization prediction were estimated using TargetP (http://www.cbs.dtu.dk/services/TargetP/) and WoLF PSORT software (http://wolfpsort.org/).

### Extraction and determination of carotenoids

The pericarp portion of tomato at 41~45 DPA (‘Micro-Tom’ Red stage) and 7 or 8 weeks DPA (‘White Beauty’ and ‘Black’) was used to extract and determine carotenoids. In ‘Black’ the pericarp portion in the upper half of the fruit was used. Identification, extraction and quantification of carotenoids have been described previously [42]. In this study, sample weights of 1.5 g for mature fruit and 3.0 g for immature fruit were used to extract carotenoids. beta-Carotene, lycopene, all-*trans*-violaxanthin, and lutein were quantified in the pulp of tomato. Carotenoid content is reported as μg g^-1^ fresh weight. Carotenoid quantification was performed in three replicates.

### Extraction and determination of flavonoids

Flavonoid samples were extracted from pericarp at same stage that isolated carotenoids. In ‘Black’, pericarp of the upper half of the fruit was used. Samples for flavonoid extraction were lyophilized and ground to a fine powder with mortar and pestle. The sample (20 mg dry weight) was extracted in 1 ml of methanol: dimethyl sulfoxide (1:1, v/v). The extract was homogenized further in a Polytron. The extract was centrifuged at 2,500*g* for 10 min and filtered using 0.2 μm Syringe filter (GLchromatodisk Kurabo).

An aliquot (2 ml) was separated by HPLC (Jasco, Tokyo, Japan) equipped with a YMC-UltraHT Pro C18 column of 100- x 3.0-mm i.d. (Waters, Milford, MA) at a flow rate of 0.6 ml min^-1^. The gradient elution schedule consisted of an initial 30 sec of 22% methanol: acetonitrile (1:1, v/v) and 78% 20 mM phosphoric acid followed by a linear gradient of 84% methanol:acetonitrile (1:1, v/v) and 16% 20 mM phosphoric acid for 47.5 min. The absorption spectrum (220 to 450 nm) of the eluate was monitored by a photodiode array detector (MD-2015 plus, Jasco, Tokyo, Japan). The peaks were identified by comparing their specific retention times and absorption spectra with authentic standards. Flavonoid content is reported as mg g^-1^ dry weight. Flavonoid quantification was performed in three replicates.

### RT-PCR (Reverse transcriptase-polymerase chain reaction)

We isolated total RNA from fruits at 41~45 DPA (‘Micro-Tom’ Red stage) and 7 or 8 weeks DPA (‘White Beauty’ and ‘Black’). First-strand cDNA was obtained using 1 μg of total RNA and the Prime Script High Fidelity RT-PCR kit (Takara Japan) according to the manufacturer’s protocol. The primer sequences of the genes used were: 5’- GGGGAATTTGGGCTTGTTGAGT -3’ (forward) and 5’- CCTTTGATTCAGGGGCGATACC -3’ (reverse) for the *Psy1* (*Phytoene synthase1*); 5’- TAACTGCCAAACCACCACAA -3’ (forward) and 5’- ACCCATTGATTCGCTACCAG -3’ (reverse) for the *Pds* (*Phytoene desaturase*); 5’- CAGAAGTGGAGGGAATTGGA -3’ (forward) and 5’- AGGCATGTAAGGGTCACCAG -3’ (reverse) for the *Zds* (*Zeta-carotene desaturase*); 5’- GATCGCCAAATCCTTAGCAA -3’ (forward) and 5’- GCCCTGGGAAGAGTGTTTTT -3’ (reverse) for the *CRTISO* (*Carotenoid isomerase*); 5’- CCCTTCACCACTCTCCATGT -3’ (forward) and 5’- TCCCTCCAATCCATAAGCAC -3’ (reverse) for the *Cyc-B* (Chromoplast-specific *lycopene beta-cyclase* **)**; 5’- AGTGGTAATCGGCTGTGGTC -3’ (forward) and 5’- GTCACAAACCTGCAGGGAAT -3’ (reverse) for the *Lcy-E* (*Lycopene epsilon-cyclase*); 5’- CCCGGGTATCAAGTTGCTTA -3’ (forward) and 5’- ACCATATAACCGGTGGATGG -3’ (reverse) for the *Lcy-B* (*Lycopene beta-cyclase*). Reaction conditions included 30 cycles of 10 sec at 98°C, 15 sec at 55°C, and 1 min at 72°C. All RT-PCR experiments were performed 3 times to confirm results, with additional semi-quantitative analysis at 28 and 29 cycles for consistency.

## Result

### Plastid differentiation of tomato fruit cells

To learn more about the relationship of fruit color and plastid differentiation, the different developmental stages of ‘Micro-Tom’, ‘Black’, and ‘White Beauty’ varieties were compared morphologically. ‘Black’ appears black due to a mix of the red color of lycopene and the green color of chlorophyll remaining on the shoulder of the fruit (Fig 1A). The frozen sections of the cuticle layers of three fruits showed that carotenoid accumulated in the epidermal and pericarp cells of ‘Micro-Tom’ (red stage) and ‘Black’, but not in ‘White Beauty’ (Fig 1B). In order to investigate the relationship between plastid morphology and the change in fruit color, fruit cells were examined with electron microscopy. Plastid morphology in pericarp tissue of ‘Micro-Tom’ (green to red developmental stages) ‘Black’, and ‘White Beauty’ were examined (Fig 1C). As a result, clear differences were found in the plastid morphology of ‘Micro-Tom’, ‘Black’ and ‘White Beauty’. Membrane structures with high molecule density (Fig 1C red arrow) and crystal structure (Fig 1C blue arrow) were observed in ‘Micro-Tom’ at the orange stage. It was also found that ‘Micro-Tom’ (red stage) has chromoplast with a whirled membrane structure (Fig 1C yellow arrow). In ‘Black’ small thylakoids similar to those found in the yellow stage of ‘Micro-Tom’ were also observed. The mature-stage chromoplast of ‘White Beauty’ looks similar to the plastid morphology of the immature stage of yellow stage ‘Micro-Tom’.

### Identification of plastid proteins using GeLCMS

Plastids were isolated using density gradients of Nycodenz (Fig 2A). To confirm that intact plastids were being isolated, they were visualized via a fluorescence microscope (Olympus SZX9) (Fig 2B). Upon examining chlorophyll autofluorescence, it was also discovered that fluorescent-red areas matched green plastid areas. The organelle were dyed with DAPI. When an organelle is damaged, DAPI enters the organelle and the DNA inside glows. However in our experiment the organelle DNA did not glow, thus confirming that the plastids were indeed intact (Fig 2B). Subcellular localization prediction programs (WoLF PSORT (http://wolfpsort.org/) and TargetP (http://www.cbs.dtu.dk/services/TargetP/)) were used to examine proteins identified, and 71% were found to be related to plastid localization (S1 Table). The fact that samples include proteins which are not expected to be plastid proteins (by subcellular localization prediction programs) is common to chromoplast proteome data in other studies [10, 11].

In order to identify as many plastid proteins as possible, GeLCMS proteomics was used. SDS-PAGE was performed on 100 μg of plastid proteins using a 12.5% poly-Acrylamide gel. Phoresis was stopped with 1/3 of the gel remaining, with the gel cut and separated into 7 sections. Proteins in the gel pieces were digested in gel, from which peptide fragments were acquired. MASCOT Search software was used to identify plastid proteins by searching MS/MS spectra against a sequence database.

A total of 605 proteins were identified from the four developmental stages of ‘Micro-Tom’ (green, yellow, orange, and red) (S1 Table). The greatest number of proteins were found in the orange maturity stage (414 in green, 385 in yellow, 446 in orange, and 310 in red, respectively). Photosynthesis proteins such as D1 protein, PSI P700 apoprotein, and RuBisCo activase were all detected through the orange maturity stage (S1 Table). This indicates that at a minimum there is plastid differentiation (the disappearing of thylakoids in chloroplast, and an increase in membrane structures with high molecular density) taking place before all photosynthesis proteins have changed to lipid and secondary metabolite synthesis proteins (Fig 1C). At the red maturity stage all photosynthesis proteins cease detection; and the total number of detected proteins decreases. Proteins related to ethylene synthesis (such as E8 protein and arginase 1) and proteins related to carotenoid synthesis (such as GcpE, Phytoene dehydrogenase, CHRC, Zeta-carotene desaturase and Carotenoid isomerase) were detected in greater numbers in the red maturity stage (S1 Table).

Plastids in the orange maturity stage have the function of both chloroplast and chromoplast. In this stage the most actively various proteins related to photosynthesis and carotenoid synthesis were accumulated. Superoxide dismutase was also detected in the orange and red developmental stages (S1 Table).

A search on the 605 chromoplast proteins was carried out for Biological Process, Molecular Function and Cellular component under Protein Ontology enrichment analysis, categorizing them by function (S1–S3 Data).

Of the 336 categories under Biological Process, we can see that while many proteins are categorized under Primary Metabolic Process, there are also many metabolites found under Carbohydrate, Carboxylic Acid, Cellular Macromolecule, and Carotenoid (S1 Data). That a large number of isolated proteins are related to Metabolic Process may be due to the fact that chromoplast contains carotenoid, which is thought to be responsible for the creation and collection of metabolites.

Plastoglobules, which play a role in plastid lipid storage and are thought to also have a role in plastid differentiation, include carotenoid synthetic enzymes [43]. 13 proteins categorized under Plastoglobules were detected, including fibrillin family proteins and plastid-lipid associated protein (S3 Data, S1 Table). 184 proteins were found to belong to Response to Stimulus, perhaps due to the increase of sugar content during fruit ripening/maturation, or other factors such as oxidative-stress defense or response to senescence. Many proteins were categorized under Transport (81 proteins) and Nitrogen Compound Related Proteins (41 proteins); this may be due to post-breaker stage photosynthesis protein degradation, nitrogen transport and reuse. Proteins related to transport are active, as products of photosynthesis in leaves are being transported to fruit.

Of the 148 categories under Molecular Function, results showed many proteins categorized under Catalytic Activity and Binding (S2 Data). The Binding category contained a large number of proteins related to nucleotide and ribonucleotide binding. It is possible that transcriptional activity is increasing to build up specific metabolites for chromoplast. The reason for this is likely due to changes during the ripening/maturation process.

Using the plprot plastid proteome database (http://www.plprot.ethz.ch/) [44] the 605 chromoplast proteins identified in ‘Micro-Tom’ were compared to plastid proteome data found in other plants (chloroplast proteins in *Arabidopsis*, proplastid proteins in tobacco, and etioplast proteins in rice). Of the 605, 48 proteins were found to be common in all four plants (S1 Fig). 82 ‘Micro-Tom’ chromoplast proteins matched with chloroplast in *Arabidopsis* (30 and 18 for proplastid proteins in tobacco and etioplast proteins in rice, respectively). 426 of the chromoplast proteins in ‘Micro-Tom’ were tomato-specific. In the 48 common proteins mentioned above, glutathione S-transferase and chloroplast elongation factor TuA were found (S1 Table). Many proteins in the green and red stages of tomato were identified as common, however the number of proteins common to chloroplast in *Arabidopsis* was fewer than expected (S1 Table). When comparing the chromoplast proteins of bell pepper (155 proteins [10]) to that of red maturity stage tomato (310 proteins), only 11 common proteins were found (lethal leaf spot 1-like protein, GcpE, phosphoglycerate kinase precursor-like protein, ATP-dependent Clp protease ATP-binding subunit clpA homologue CD4B, transaldolase, thioredoxin peroxidase, harpin binding protein-1, Hsp70, Elongation factor 1-alpha, Leucine aminopeptidase and other unknown proteins) (S1 Table). The reason there are so few common proteins may be due to the fact that many of the proteins in bell pepper are related to capsaicin synthesis [10]. When comparing our results to the tomato proteome data of Barsan et al. [11], 58% of chromoplast proteins at red maturity stage were common. The biggest difference was that there was only one protein in our chromoplast proteome data related to Toc/Tic complexes/protein import systems. Also, for proteins over 105kDa and less than 20kDa, there were even fewer similar proteins between our case and the Barsan study [11] (S1 Table). While in the same family *Solanaceae*, the chromoplast proteomes of bell pepper and tomato have very few common proteins. Only 32% of the 605 proteins isolated from the 4 stages in our study were common to the data in Barsan et al. [11]–this is likely due to differing MS analysis conditions, or even slightly different timing during ripening/maturation between fruit samples.

### Carotenoid and flavonoid content by tomato color

**Fig 3:**
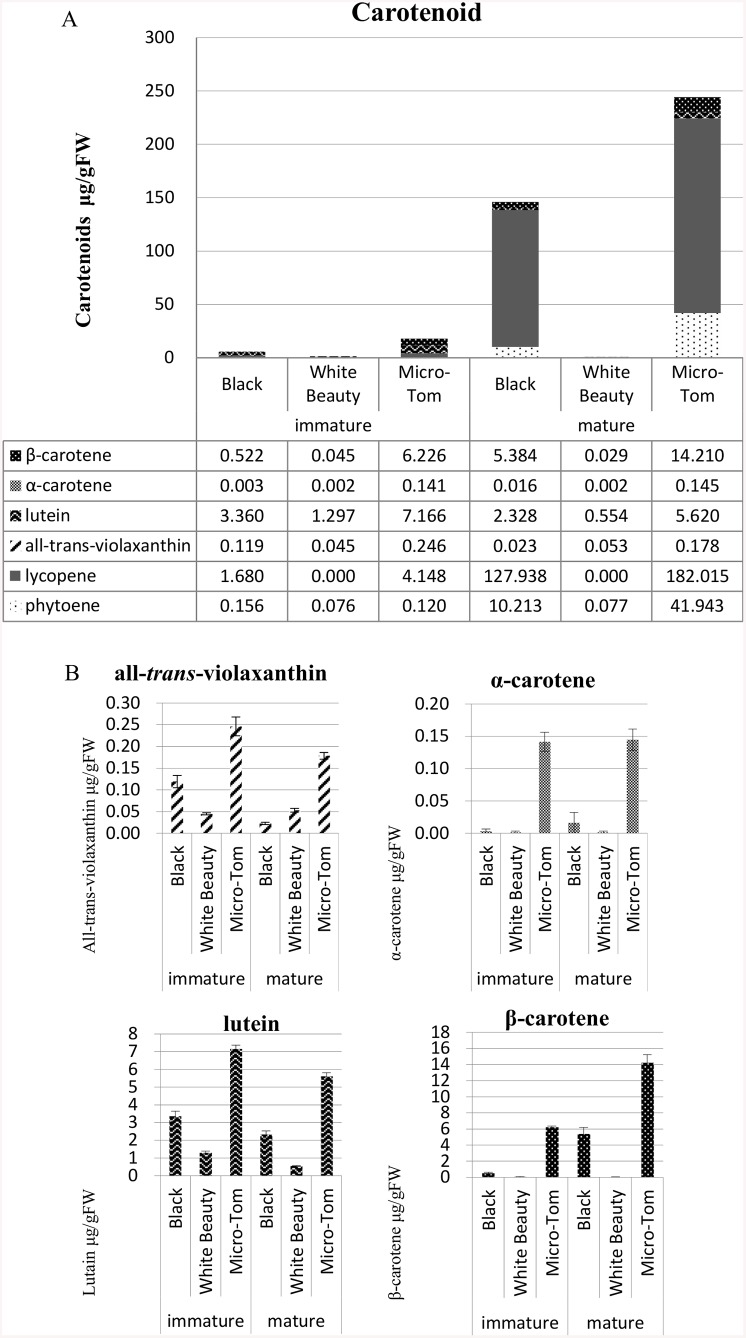
Carotenoid content in ‘Black’, ‘White Beauty’ and ‘Micro-Tom’ (Red). A. Total amount of carotenoids measured in (both) immature and mature fruits. B. Amount of all-*trans*-violaxanthin, α-carotene, lutein and β-carotene in (both) immature and mature fruits. Sample weights of 1.5 g for mature fruit and 3.0 g for immature fruit were used to extract carotenoids. beta-Carotene, lycopene, all-*trans*-violaxanthin, and lutein were quantified in the pulp of tomato. Carotenoid content is reported as μg g^-1^ fresh weight. Carotenoid quantification was performed in three replicates.

We studied whether or not difference in fruit color is related to the amount of carotenoid present (Fig 3), and using our methods found that ‘White Beauty’ had virtually undetectable levels of carotenoid. RT-PCR was used to examine the mRNA accumulation of genes related to carotenoid synthesis such as *PSY1* (*Phytoene synthase1*), *PDS* (*Phytoene desaturase*), *ZDS* (*Zeta-carotene desaturase*), *CRTISO* (*Carotenoid isomerase*), *CYC-B* (Chromoplast-specific *lycopene beta-cyclase*), *LCY-E* (*Lycopene epsilon-cyclase*), and *LCY-B* (*Lycopene beta-cyclase)* in ‘Micro-Tom’ (red maturity stage), ‘White Beauty’, and ‘Black’, but significant differences of mRNA accumulation were not found (S2 Fig).

**Fig 4:**
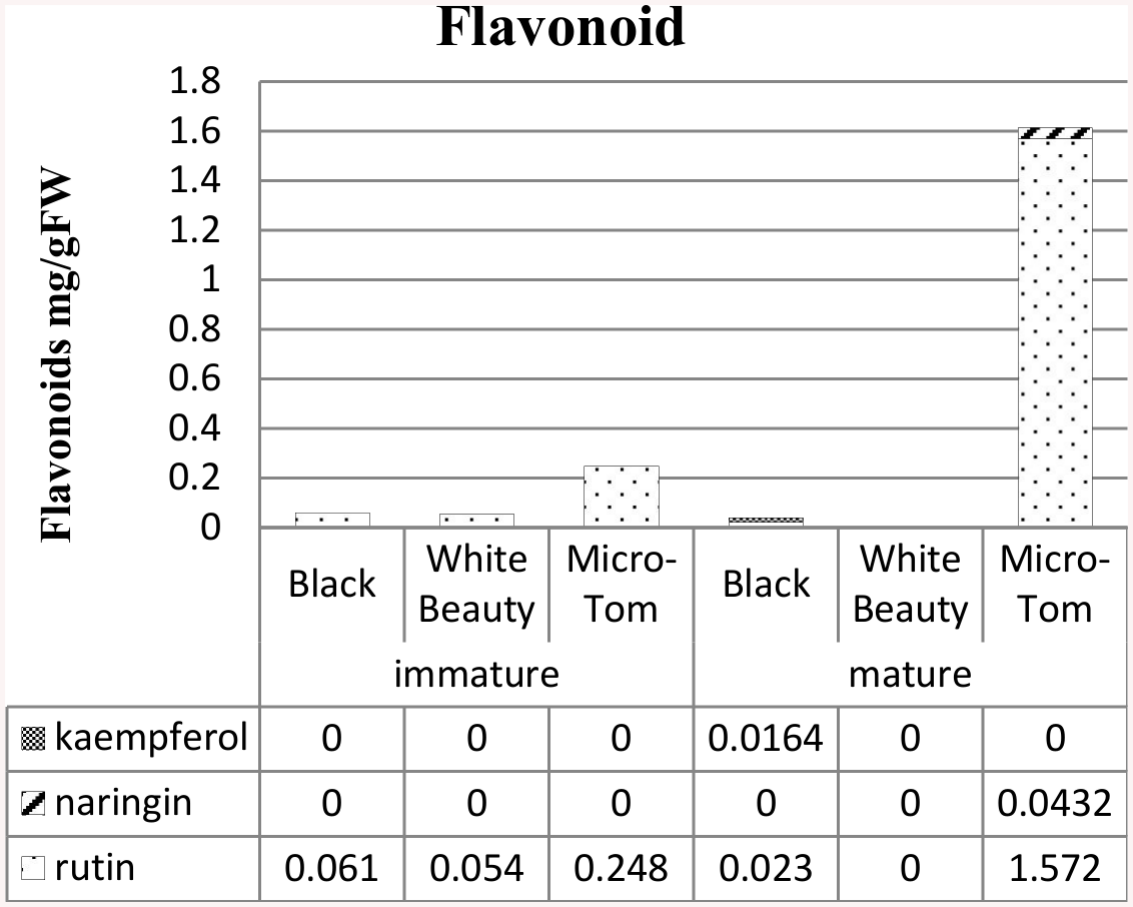
Flavonoid content in ‘Black’, ‘White Beauty’ and ‘Micro-Tom’ (Red). The sample was 20mg (dry weight) of the pericarp portion. Flavonoid content is reported as mg g^-1^ dry weight. Flavonoid quantification was performed in three replicates.

We studied whether or not difference in fruit color is related to the amount of flavonoid present (Fig 4). Mature ‘White Beauty’ also contained no flavonoid content (Fig 4).

### Chromoplast protein accumulation by tomato color using 2D-gel electrophoresis

**Fig 5:**
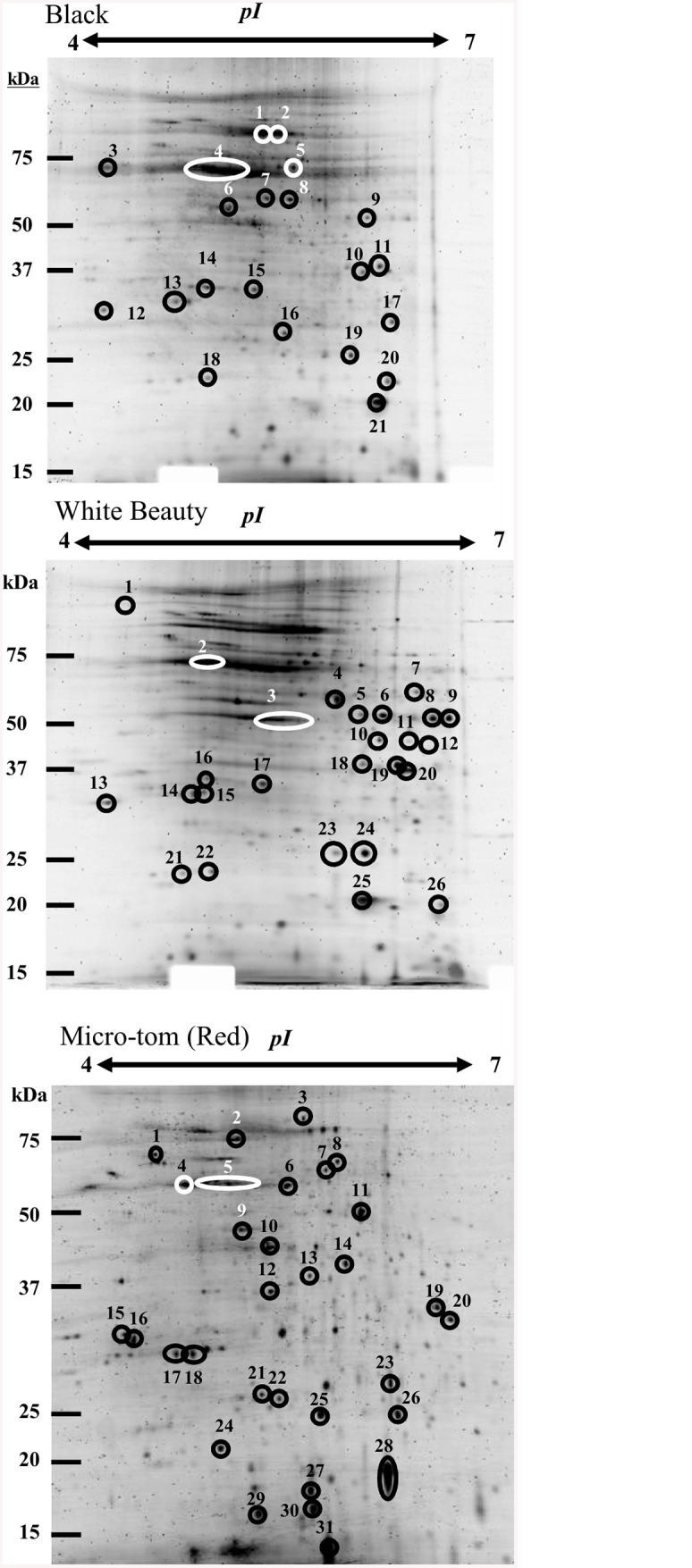
2D gel electrophoresis of chromoplast proteins in fruit cells of ‘Micro-Tom’ (Red), ‘Black’ and ‘White Beauty’. Using an 11 cm IPG DryStrip (pH4-7), 100 μg of solubilized proteins were separated. Two-dimensional gel electrophoresis was performed with 12% poly-Acrylamide gel (16 cm x 16 cm). Following electrophoresis, the gel was stained with Flamingo Fluorescent Gel Stain (BIO-RAD). A list of proteins numbered in Fig 5 can be found in Table 1.

In order to understand the connection of fruit color to changes in plastid proteome, a comparison of the chromoplast proteome data of ‘Micro-Tom’, ‘Black’ and ‘White Beauty’ using 2D-gel electrophoresis was performed. A 2D-PAGE of the red maturity stage of ‘Micro-Tom’ showed that proteins were detected from high to low molecular weight (Fig 5). 2D-PAGE of the mature stage of ‘Black’ was different from the red maturity stage of ‘Micro-Tom’ in that ‘Black’ had many proteins related to photosynthesis.

**Fig 6:**
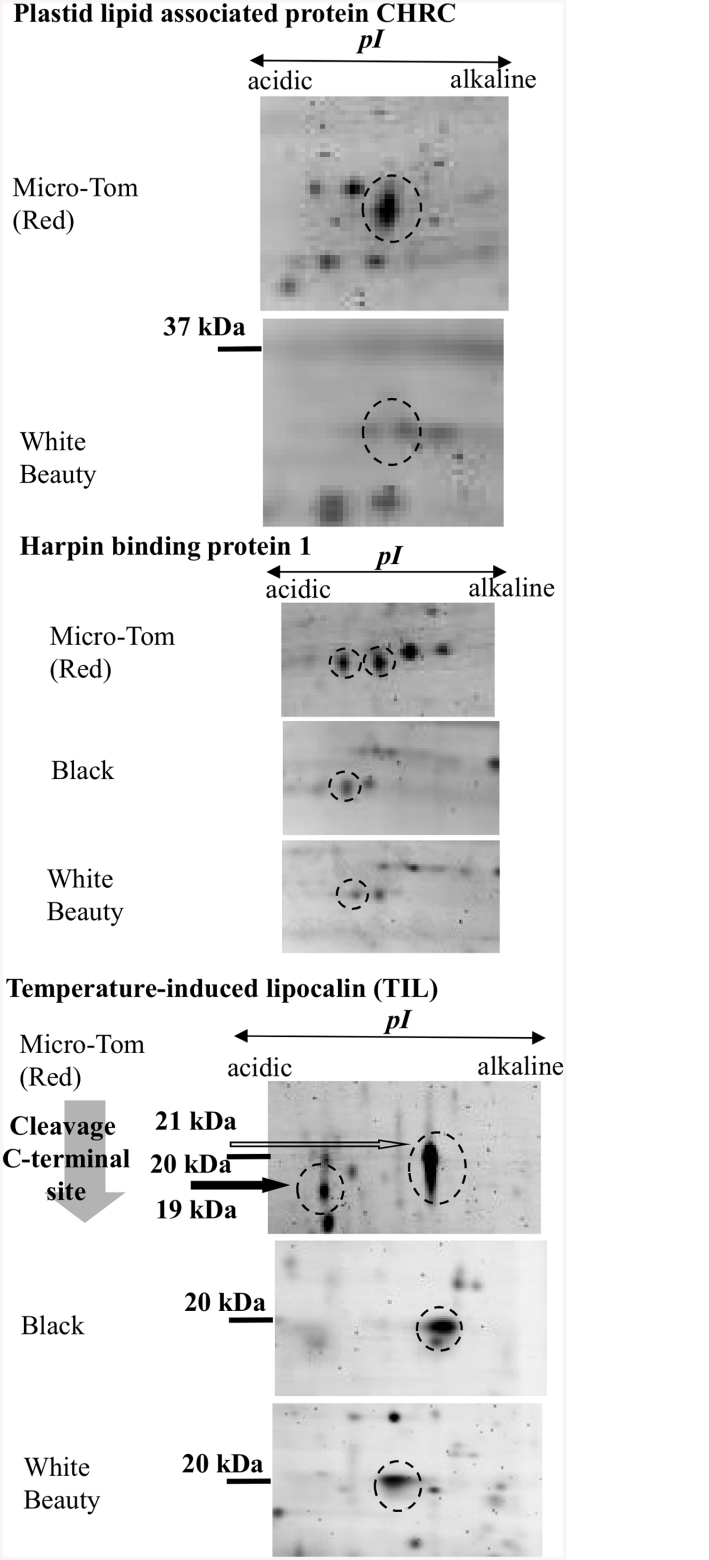
Proteins of varying levels of accumulation in ‘Micro-Tom’ (Red), ‘Black’ and ‘White Beauty’. Enlarged images of 2D-PAGE results from Fig 5 that showed significant differences in protein accumulation. Protein spots of note are indicated with broken line circles. CHRC: ‘Micro-Tom’ spot16, ‘White Beauty’ spot16, HrBP1: ‘Micro-Tom’ spots17 and 18, ‘Black’ spot13, ‘White Beauty’ spot14, TIL: ‘Micro-Tom’ spots27 and 28, ‘Black’ spot 21, ‘White Beauty’ spot25.

The plastid lipid associated protein (CHRC) spots for ‘Micro-Tom’ and ‘White Beauty’ were different (Fig 6). CHRC is a protein related to carotenoids that exists uniquely in plastid [45–47]. As it is accumulated in the red maturity stage of ‘Micro-Tom’ to the extent that it is visible in 2D-PAGE, it is thought that this protein may play a major role in chromoplast differentiation and in carotenoid accumulation [45, 47].

Harpin binding protein-1 (HrBP1) and temperature-induced lipocalin (TIL) were found as spots in the plastids of all three varieties (Fig 6). HrBP1 in *Arabidopsis* is a member of the PAP/fibrillin sub-family, and has lipocalin-like characteristics. Two spots were detected in the red maturity stage of ‘Micro-Tom’, while only one spot was found in the results for ‘Black’ and ‘White Beauty’ (respectively).

Similarly, TIL results varied between varieties. ‘Black’ showed a large spot on the alkaline isoelectric point of the spectrum, while ‘White Beauty’ had a stronger spot on the acidic isoelectric point (Fig 6). Not only were the isoelectric points different for TIL, their mass was also different. 2D-PAGE results show what looks to be a cleaved C-terminal, with 21kDa of TIL proteins becoming 19kDa (‘Micro-Tom’ red maturity stage). A similar phenomenon was reported in a study on TIL in wheat and *Arabidopsis*, where it was found that they have a putative C-terminal cleavage site [18, 19]. Considering this cleavage site, the calculated molecular mass of mature TIL proteins would be 2kDa smaller than the corresponding precursor.

## Discussion

### Plastid proteins and tomato fruit development

Similar to the proteomic analysis by Barsan et al., in this study there were fewer identified proteins in the red maturity stage when compared to other color/maturity stages (S1 Table) [11]. Barsan et al. analysed the plastidic proteomes of mature green, breaker, and red stages [12]. We analyzed and were able to acquire plastid proteome data from 4 stages—green, yellow, orange, and red—which lead us to discover that thylakoid proteins involved in photosynthesis such as D1, D2, and OEE1 remain through the orange maturity stage (S1 Table). Results showed that RuBisCo (an enzyme involved in carbon dioxide fixation in chloroplast) was detected through the red maturity stage (similar to results in Barsan et al. [12]). Why RuBisCo remains even though photosynthesis is no longer taking place is a mystery. It is possible that RuBisCo remains in fruit cells as a nitrogen source.

GeLCMS proteomics analysis showed that photosynthesis proteins including Cytochrome b6f complex and Chlorophyll a-b binding protein were accumulated in the green maturity stage of ‘Micro-Tom’. As the electron micrographs in Fig 1C show, grana stacked thylakoids are present in green maturity stage chloroplast, giving it a look resembling that of chloroplast isolated from leaves in *Arabidopsis* [48]. As mentioned above, 82 proteins were found to be common between ‘Micro-Tom’ (all four developmental stages) and leaf *Arabidopsis* chloroplast—indicating that while their morphologies look similar, their functions may be quite different. It is known that photosynthesis in fruit is very different from that of leaves [49–51]. Photic saturation points in tomato fruits generally tend to hover around 15~25 klx, with smaller values in young fruit, growing larger as fruit mature. In leaves however the opposite is true—young leaves have higher photic saturation points, decreasing with age. Stomata are present in the epidermis of leaves and fruits, with a significantly higher number found in leaves. In fruit, the number of stomata is set at anthesis and remains constant throughout fruit development, so as the surface of fruit expands during growth, stomatal frequency decreases [49]. Various heat shock and lipoxygenase proteins were detected and detection increased with fruit ripening (S1 Table). The reason for this could be due to heat dissipation becoming more difficult as stomatal frequency decreases.

It is also reported that the chlorophyll *a*/*b* ratio in the early stages of tomato fruit development is 2.44, but in the later stages of development it is below 1.0, indicating an increase in chlorophyll *b* and the absorption of low light [50]. Photosynthesis on the surface of fruit requires a supply of CO_2_ (originating internally in fruit), and it is thought that photosynthetic oxygen supplements the low internal oxygen levels of fruit [51]. These and other findings confirm that there are a number of differences between leaf photosynthesis and the internal energy mechanisms of fruit.

Geranylgeranyl reductase (a protein related to carotenoid synthesis) was also detected. Fleshy fruits have depth and as such, light can only penetrate to a certain degree [52, 53]. The presence of geranylgeranyl reductase could indicate that carotenoids are functioning internally to capture light that chlorophyll does not originally absorb on the surface [52, 53].

Moreover, the development of chromoplast is accompanied by increased carotenoid synthesis. Carotenoid accumulation brings about substantial changes in the shape, morphology, and internal structure of plastids (Fig 1) [43, 54, 55]. The formation of carotenoid-rich membranous sacs, increase in number and size of plastoglobules, and the appearance of carotenoid-containing crystalloids are characteristic of chromoplast development [43]. However, proteins related to photosynthesis were detected throughout the orange maturity stage. By nature fruit have a higher rate of respiration and a negative rate of photosynthesis—the photosynthesis proteins remaining in the orange maturity stage may have some function in balancing the rate of respiration and slowing the decrease in rate of photosynthesis. While there was a significant and sudden decrease in photosynthesis proteins in the red maturity stage, from the yellow maturity stage onward there was an increase in the detection of E8 proteins and arginase related to ethylene synthesis. There was also an increase in GcpE, Phytoene dehydrogenase, CHRC, zeta-carotene desaturase and Carotenoid isomerase, proteins all related to carotenoid synthesis. It can be seen from carotenoid accumulation and changes in plastid morphology that proteins related to carotenoid synthesis are being accumulated at relatively early stages of maturity (Figs 1C and 3).

Superoxide dismutase and heat shock proteins were found in ‘Micro-tom’ from the orange maturity stage onward. Superoxide dismutase is known to aid in the reduction of harmful reactive oxygen species (ROS). There have been many other ROS-related proteins detected in other tomato chromoplast [11, 12]. TIL protein is reported to be related to oxidative stress [56]. An AtTIL overexpressing transgenic plant has enhanced tolerance to stress caused by freezing, Paraquat and light [56]. Also, while pepper fruit ripens, superoxide dismutase and proteins of the ascorbate-gluthathione cycle increase [57]. From these results it can be assumed that the reduction of ROS is a key factor in fruit and chromoplast development [58]. It is thought that as fruits mature, glucose accumulates in cells and intercelluar osmotic pressure rises, giving way to ROS formation. Superoxide dismutase, TIL, and heat shock proteins may play some role in suppressing ROS formation.

### Plastid proteins and fruit color

‘Black’ tomato plastids (mature stage) have thylakoids (Fig 1C). Due to the detection of photosynthetic water oxidation complex 33kDa, differentiation from chloroplast to chromoplast that would normally occur during the fruit maturation process may have stopped (S1 Table). This would also explain perhaps the green color areas on the shoulder of ‘Black’ tomato fruit.

While ‘White Beauty’ had virtually undetectable levels of carotenoid (Fig 3), significant differences of expression of carotenoid synthesis genes were not detected in ‘White Beauty’ when compared to ‘Micro-tom’ and ‘Black’ (S2 Fig). In the *psy1* mutant (line 5381; W180*), a point mutation in the *PSY1* gene did not stop the accumulation of *PSY1* mRNA, however proteins were not accumulated, and carotenoid synthesis did not take place [59]. A similar point mutation may be responsible for this phenomenon in ‘White Beauty’. Mature ‘White Beauty’ also contained no flavonoid content (Fig 4). There are reports of high levels of narigenin chalcone accumulation in a yellow-colored natural mutant called *yellow flesh* mutant [60, 61], but it is rare to find this phenomenon among non-processed cultivars.

CHRC, HrBP1, and TIL were found to accumulate differently in ‘Micro-Tom’ (red maturity stage), ‘Black’ and ‘White Beauty’ (Fig 6). CHRC was reported to be localized in chromoplast [45]. It has an amino acid sequence similar to fibrillin and accumulates in the plastids of senescing leaves [45]. CHRC is involved in carotenoid accumulation and stabilization [45]. Induction of CHRC and fibrillin develops fibrils and causes carotenoids to accumulate in chromoplast [45]. It has been reported that when down regulating the expression of CHRC in tomato, the accumulation of carotenoid in tomato flower decreases by 30% [62]. A comparison of 2D-PAGE data showed that while ‘White Beauty’ contained CHRC, it was at a smaller amount/quantity than that of ‘Micro-Tom’ (red maturity stage)(Fig 6 and Table 1). GeLCMS proteomics data showed that CHRC was detected from the yellow maturity stage onward. These results indicate that CHRC plays a key role in carotenoid accumulation and chromoplast differentiation (S1 Table).

**Table 1:** Protein list of the 2D-PAGE numbering spots in Fig 5.

Black							
Spot number^a^	Protein^b^	gi	Mass	Score^c^	Matched^d^	Sequence cover (%)	Species
1	NAD-dependent malic enzyme 62 kDa isoform	gi|585451	69909	601	8	20	Solanum tuberosum
	succinate dehydrogenase 1–1	gi|297797713	69599	246	6	12	Arabidopsis lyrata subsp. lyrata
2	hypothetical protein	gi|225428336	52274	172	3	6	Vitis vinifera
3	Calreticulin	gi|11131769	47452	161	3	10	Nicotiana plumbaginifolia
	ATP synthase F1 subunit 1	gi|57013987	55191	323	9	19	Nicotiana tabacum
	nucleotide-binding subunit of vacuolar ATPase	gi|166627	54705	53	1	3	Arabidopsis thaliana
	F1-ATPase alpha subunit	gi|113197031	44751	203	6	15	Epipactis helleborine
4	ATP synthase subunit beta	gi|114557	53437	134	4	9	Nicotiana rustica
	ATP synthase CF1 beta chain	gi|89280642	53434	1393	7	52	Solanum lycopersicum
5	mitochondrial processing peptidase-like	gi|82621176	58070	479	7	24	Solanum tuberosum
	ATP synthase F1 subunit 1	gi|57013987	55191	323	9	19	Nicotiana tabacum
	F1-ATPase alpha subunit	gi|113197031	44751	203	6	15	Epipactis helleborine
6	actin	gi|3219762	37464	335	6	27	Nicotiana tabacum
7	polygalacturonase-2	gi|129939	50020	150	4	11	Solanum lycopersicum
8	predicted protein	gi|224116582	50690	142	6	14	Populus trichocarpa
9	elongation factor tu	gi|255567660	49002	589	7	28	Ricinus communis
	formate dehydrogenase	gi|26454627	42012	89	4	13	Solanum tuberosum
10	putative vacuolar proton ATPase subunit E	gi|9652289	27114	298	3	21	Solanum lycopersicum
11	beta-cyanoalanine synthase like protein	gi|11995005	37611	738	6	41	Solanum tuberosum
	cysteine synthase	gi|255555901	39424	140	3	13	Ricinus communis
12	progesterone-binding protein homolog	gi|4960154	28210	154	1	13	Arabidopsis thaliana
13	harpin binding protein 1	gi|38679319	30065	194	4	30	Solanum lycopersicum
14	chloroplast photosynthetic water oxidation complex 33kDa subunit precursor	gi|152143640	28249	97	3	17	Morus nigra
15	remorin 1	gi|4731573	21837	79	3	21	Solanum lycopersicum
16	hypothetical protein	gi|147819925	27678	45	1	3	Vitis vinifera
17	hypothetical protein	gi|147819925	27678	45	1	3	Vitis vinifera
18	ATP synthase D chain	gi|48209968	19795	237	5	42	Solanum demissum
	40S ribosomal protein S7-like protein	gi|76160947	22100	66	2	8	Solanum tuberosum
19	superoxide dismutase	gi|134672	25488	137	3	17	Nicotiana plumbaginifolia
20	small heat shock protein	gi|22530880	25712	113	3	13	Solanum lycopersicum
21	temperature-induced lipocalin	gi|77744873	21452	72	5	42	Solanum lycopersicum
White Beauty							
Spot number	Protein	gi	Mass	Score	Matched	Sequence cover (%)	Species
1	rubisco subunit binding-protein alpha subunit (60 kDa chaperonin alpha subunit)	gi|31193919	61363	550	7	16	Oryza sativa Japonica Group
2	glutamine synthetase GS1	gi|209529862	38527	431	7	29	Solanum tuberosum
3	glutamine synthetase GS1	gi|209529862	38527	431	7	29	Solanum tuberosum
4	alcohol dehydrogenase 1	gi|113365	41068	72	3	7	Solanum tuberosum
5	elongation factor tu	gi|255567660	49002	754	7	31	Ricinus communis
	NADP-dependent isocitrate dehydrogenase-like protein	gi|3687404	43136	290	3	15	Solanum lycopersicum
6	elongation factor tu,	gi|255567660	49002	754	7	31	Ricinus communis
	NADP-dependent isocitrate dehydrogenase-like protein	gi|3687404	43136	290	3	15	Solanum lycopersicum
	pyruvate dehydrogenase	gi|12003246	43346	106	3	8	Solanum lycopersicum
	formate dehydrogenas	gi|26454627	42012	763	7	39	Solanum tuberosum
7	SH3 domain-containing protein	gi|297798448	40949	133	3	8	Arabidopsis lyrata subsp. lyrata
	fumarase	gi|1488652	53348	79	2	4	Solanum tuberosum
8	formate dehydrogenase	gi|26454627	42012	763	7	39	Solanum tuberosum
9	Nad-dependent formate dehydrogenase	gi|4760553	41217	82	2	4	Oryza sativa
10	putative mitochondrial NAD-dependent malate dehydrogenase	gi|21388544	36172	242	4	19	Solanum tuberosum
11	unknown	gi|77745499	34789	429	5	23	Solanum tuberosum
12	ATP synthase gamma chain chloroplastic	gi|231610	41421	193	4	14	Nicotiana tabacum
	chloroplast cysteine synthas	gi|327493149	29448	78	2	7	Solanum nigrum
	beta-cyanoalanine synthase	gi|11995005	37611	239	5	34	Solanum tuberosum
13	putative progesterone-binding protein homolog	gi|4960154	28210	186	1	13	Arabidopsis thaliana
14	14-3-3protein	gi|26454609	28796	87	2	10	Solanum lycopersicum
	harpin binding protein	gi|38679319	30065	518	5	42	Solanum lycopersicum
15	14-3-3protein	gi|26454611	29413	71	3	13	Solanum lycopersicum
16	plastid lipid associated protein CHRC	gi|350539549	35628	179	3	15	Solanum lycopersicum
	14-3-3protein	gi|26454611	29413	82	3	13	Solanum lycopersicum
17	14-3-3protein	gi|26454611	29413	82	3	13	Solanum lycopersicum
	remorin 1	gi|4731573	21837	283	7	39	Solanum lycopersicum
18	beta-cyanoalanine synthase like protein	gi|11995005	37611	239	5	34	Solanum tuberosum
19	beta-cyanoalanine synthase like protein	gi|11995005	37611	239	5	34	Solanum tuberosum
20	vacuolar ATPase subunit E	gi|29290712	25801	58	4	25	Phaseolus acutifolius
21	mitochondrial small heat shock protein	gi|3492854	23818	81	2	9	Solanum lycopersicum
22	ATP synthase D chain	gi|48209968	19795	59	3	17	Solanum demissum
23	superoxide dismutase	gi|134672	25488	48	2	8	Nicotiana plumbaginifolia
24	Superoxide dismutase	gi|134672	25488	56	2	8	Nicotiana plumbaginifolia
25	temperature-induced lipocalin	gi|77744873	21452	241	3	27	Solanum lycopersicum
26	Hop-interacting protein TH113	gi|525314283	37283	141	3	12	Solanum lycopersicum
Micro-Tom (Red maturity stage)							
Spot number	Protein	gi	Mass	Score	Matched	Sequence cover (%)	Species
1	rubisco subunit binding-protein alpha subunit	gi|84468288	61187	136	4	9	Trifolium pratense
2	vacuolar H+-ATPase	gi|131573315	68876	203	6	10	Malus domestica
3	NADH dehydrogenase [ubiquinone] iron-sulfur protein 1, mitochondrial	gi|3122572	79919	173	3	6	Solanum tuberosum
4	vacuolar H+-ATPase B subunit	gi|6715512	53861	706	9	32	Nicotiana tabacum
5	ATP synthase subunit beta	gi|114421	59819	412	6	25	Nicotiana plumbaginifolia
6	mitochondrial-processing peptidase subunit alpha	gi|266567	54643	824	7	27	Solanum tuberosum
7	mitochondrial processing peptidase-like	gi|82621176	58070	184	5	11	Solanum tuberosum
8	cytochrome c reductase-processing peptidase subunit I, MPP subunit I, P55	gi|410633	59896	350	4	19	Solanum tuberosum
9	actin	gi|3219762	37464	235	6	30	Nicotiana tabacum
10	glutamine synthetase	gi|541632	18735	226	4	32	Solanum lycopersicum
11	hop-interacting protein THI113	gi|365222922	37283	110	3	11	Solanum lycopersicum
12	annexin	gi|350538805	35894	374	7	31	Solanum lycopersicum
13	annexin	gi|350538735	36154	139	3	16	Solanum lycopersicum
14	hop-interacting protein THI113	gi|365222922	37283	74	3	11	Solanum lycopersicum
15	ripening regulated protein DDTFR10	gi|350534548	22180	95	3	36	Solanum lycopersicum
16	plastid lipid associated protein CHRC	gi|350539549	35628	179	3	15	Solanum lycopersicum
17	harpin binding protein 1	gi|350535623	30065	358	4	25	Solanum lycopersicum
18	harpin binding protein 1	gi|350535623	30065	426	4	25	Solanum lycopersicum
19	beta-cyanoalanine synthase like protein	gi|11995005	37611	195	3	15	Solanum tuberosum
20	vacuolar ATP synthase subunit E	gi|29290712	26482	230	4	17	Phaseolus acutifolius
21	ferritin	gi|116519130	28027	82	2	7	Lycoris aurea
22	unknown	gi|78191402	28009	71	2	9	Solanum tuberosum
23	carbonic anhydrase	gi|350540662	28908	430	7	49	Solanum lycopersicum
24	ATP synthase D chain	gi|48209968	19795	110	3	18	Solanum demissum
25	putative quinone reductase	gi|37724581	17612	131	2	16	Vitis vinifera
26	COSII_At3g10920	gi|224980294	3045	182	1	57	Solanum cheesmaniae
27	temperature-induced lipocalin	gi|350539735	21452	312	6	44	Solanum lycopersicum
28	temperature-induced lipocalin	gi|350539735	21509	545	6	44	Solanum lycopersicum
29	17.8 kDa class heat shock protein	gi|232273	17750	71	3	22	Solanum lycopersicum
30	17.8 kDa class heat shock protein	gi|232273	17750	71	3	22	Solanum lycopersicum
31	unknown protein 18	gi|205830697	1393	64	1	100	Pseudotsuga menziesii

^a^ Spot number as assigned in 2D-gel (Fig 5),

^b^ Protein assignment based on LC-MS/MS identification

^c^ Mascot score,

^d^ Number of peptides matched in MS analysis

Proteins in the Lipocalin family are found widely in bacteria, invertebrates, vertebrates and plants. Plant lipocalin are divided into three families: temperature-induced lipocalin, chloroplastic lipocalin (CHL) and lipocalin-like protein (violaxanthin de-epoxidase [VDS], zeaxanthin epoxidase [ZEP]) [19, 63]. Lipocalin has various functions including those related to environmental stress response, apoptosis induction, membrane formation and fixation, regulation of immune response, cell growth, and metabolism adjustment [64].

HrBP1 was reported to be localized in chromoplast. This protein plays a central role in pathogen resistance. *Arabidopsis* HrBP1 has a similar protein sequence to proteins of the PAP/fibrillin family. PAP/fibrillin family proteins have characteristics and functions similar to lipocalin, including the transport of hydrophobic molecules [65]. It is believed that lipocalin family and related proteins such as HrBP1 are related to chromoplast differentiation. Moreover, whirlpool-like structures were found in many ‘Micro-Tom’ (red maturity stage) chromoplasts (Fig 1C yellow arrow), compared to very few in the chromoplasts of ‘White Beauty’ and ‘Black’. These structures may be related in some way to HrBP1 amounts (Figs 1C and 6). The fibrillin over-expressed transgenic tomatoes showed a zone with a newly synthesized membrane network including carotenoid crystals typical of chromoplasts and increased carotenoid content [66]. Spots of TIL and HrBP1 on 2D-PAGE gel showed different MW and *pI* among ‘Micro-Tom’ (red maturity stage), ‘Black’ and ‘White Beauty’. ‘Micro-Tom’ (red maturity stage) and ‘Black’ accumulate carotenoids, but ‘White Beauty’ does not. Thus it is possible that cultivars that accumulate carotenoids have different posttranslational modification than those that do not (in this case, ‘White Beauty’). It is worth noting that TIL protein in humans is a phosphoprotein as well as a glycoprotein [67]. Differences in isoelectric points and mass of TIL proteins may be due to post-translational modification of proteins i.e. phosphorylation or glycosylation.

It is known that glycosylated lipocalin is found in humours such as human tears or saliva [67]. A search was also performed using NetGlycate (http://www.cbs.dtu.dk/services/NetGlycate/) and Plotter (http://wlab.ethz.ch/protter/start/) to look into the possibility of TIL glycosylation in tomato, and TIL were found to have predicted glycosylated sites (data not shown).

It is possible that plant TIL is glycosylated in a manner similar to how it is glycosylated in animals [65]; however further study into the posttranslational modification of tomato is necessary.

It is of worth to note that lipocalin works to transport small hydrophobic molecules, and that there are different posttranslational modification patterns found in different colors of tomato (Fig 6). It is possible that, because HrBP1 seems to have characteristics similar to the PAP/fibrillin family of proteins and because this family of proteins is related to the transport of hydrophobic molecules, that lipocalin may be related to the transport of carotenoids, (also hydrophobic) [45, 62]. It is also possible that carotenoid synthesis and accumulation act as a signal, which in turn cause lipocalin to become active through its posttranslational modification, which subsequently plays a role in transporting carotenoids to chromoplast membranes.

Lipocalins could also play a role in carotenoid synthesis similar to how Lipocalin-like-proteins Violaxanthin de-epoxidase (VDE) and Zeaxanthin epoxidase (ZE) do. VDE is an enzyme which acts as a catalyst for the conversion of Violaxanthin-> Antheraxanthin-> Zeaxanthin, while ZE works as a catalyst for conversion in the opposite direction. Lipocalin content peaks in the orange maturity stage of ‘Micro-Tom’–further indicating a possible and likely connection to carotenoid accumulation and content, which deserves further investigation.

In this study ‘Micro-Tom’, ‘Black’ and ‘White Beauty’ were used as material, with GeLCMS proteomics and 2D-PAGE as primary methods of obtaining and analyzing data. This combination allows observation of not only the increase and decrease of proteins, it also allows for the observation of proteins that may be undergoing posttranslational modification. A natural next step in the immediate future for this research is continued study into which proteins undergo posttranslational modification, and also how posttranslational modification of proteins may be linked to fruit maturity and fruit color and ultimately plastid differentiation.

## Supporting Information

S1 Data Categorization of plastid proteins by biological process. (XLSX)

S2 Data Categorization of plastid proteins by molecular function. (XLSX)

S3 Data Categorization of plastid proteins by cellular component. (XLSX)

S1 Fig Comparison of the Chromoplast proteome of ‘Micro-Tom’ to that of other plastid proteomes. ‘Micro-Tom’ chromoplast proteins isolated in this study were compared using the plprot database with the proteome analyses of other plastids, such as the chloroplast of Arabidopsis, proplastid of Tobacco, and the etioplast of Rice.
(TIF)

S2 Fig Expression analysis of carotenoid synthesis genes in red stage of ‘Micro-Tom’, ‘White Beauty’ and ‘Black’. Left side shows the carotenoid synthesis pathway. Right side shows the results of RT-PCR, with gene names printed on the left.
(TIF)

S1 Table List of the 605 plastid proteins identified via GeLCMS analysis. green*, yellow, orange, red indicate the fruit maturity stages of ‘Micro-tom’. To increase confidence of the results of MASCOT search, the results were filtered using Scaffold’s probability. % indicates the probability of accuracy of protein identification. Cells with figures indicate that said protein was detected at the maturity stage shown. WoLF PSORT and TargetP are both software used to predict subcellular localization. ‘Micro-Tom’ fruit chromoplast proteins isolated in the this study were compared with the plastid proteome data from a study by Barsan et al. [11] as well as the plprot plastid protein database web site (http://www.plprot.ethz.ch/), with matches indicated by a ○. UNKNOWN refers to chromoplast protein data from Barsan et al. [11] that lacked Arabidopsis gene annotations, and as such were not included in our comparison.
(XLSX)
