# Supplementary figures and images for: Plastid Proteomic Analysis in Tomato Fruit Development
Source: PLoS One. 2015 Sep 15;10(9):e0137266. doi: 10.1371/journal.pone.0137266 (PMC4570674; doi:10.1371/journal.pone.0137266)

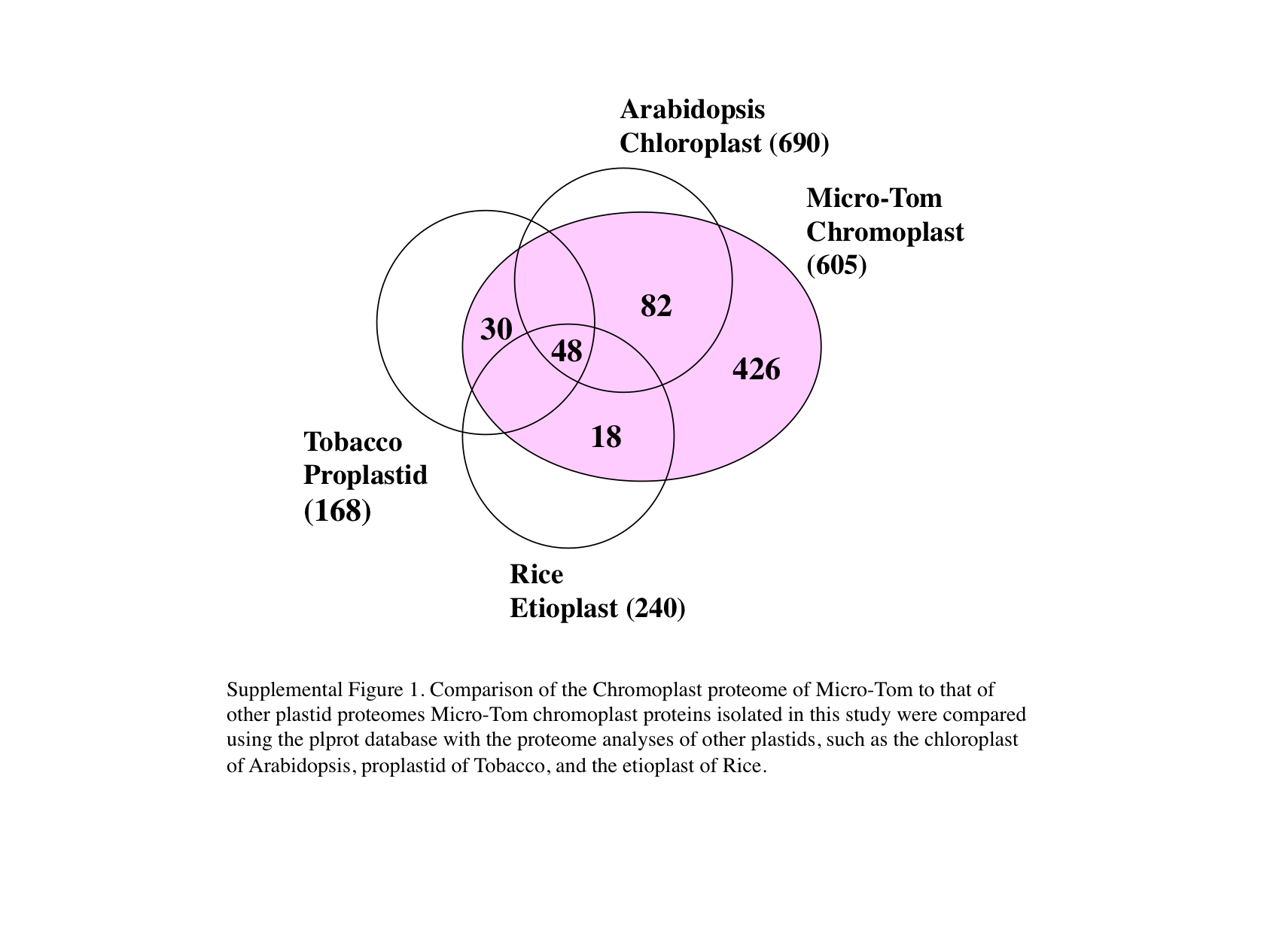

Supplement: S1 Fig — ‘Micro-Tom’ chromoplast proteins isolated in this study were compared using the plprot database with the proteome analyses of other plastids, such as the chloroplast of Arabidopsis, proplastid of Tobacco, and the etioplast of Rice. (TIF) [file pone.0137266.s004.tif]

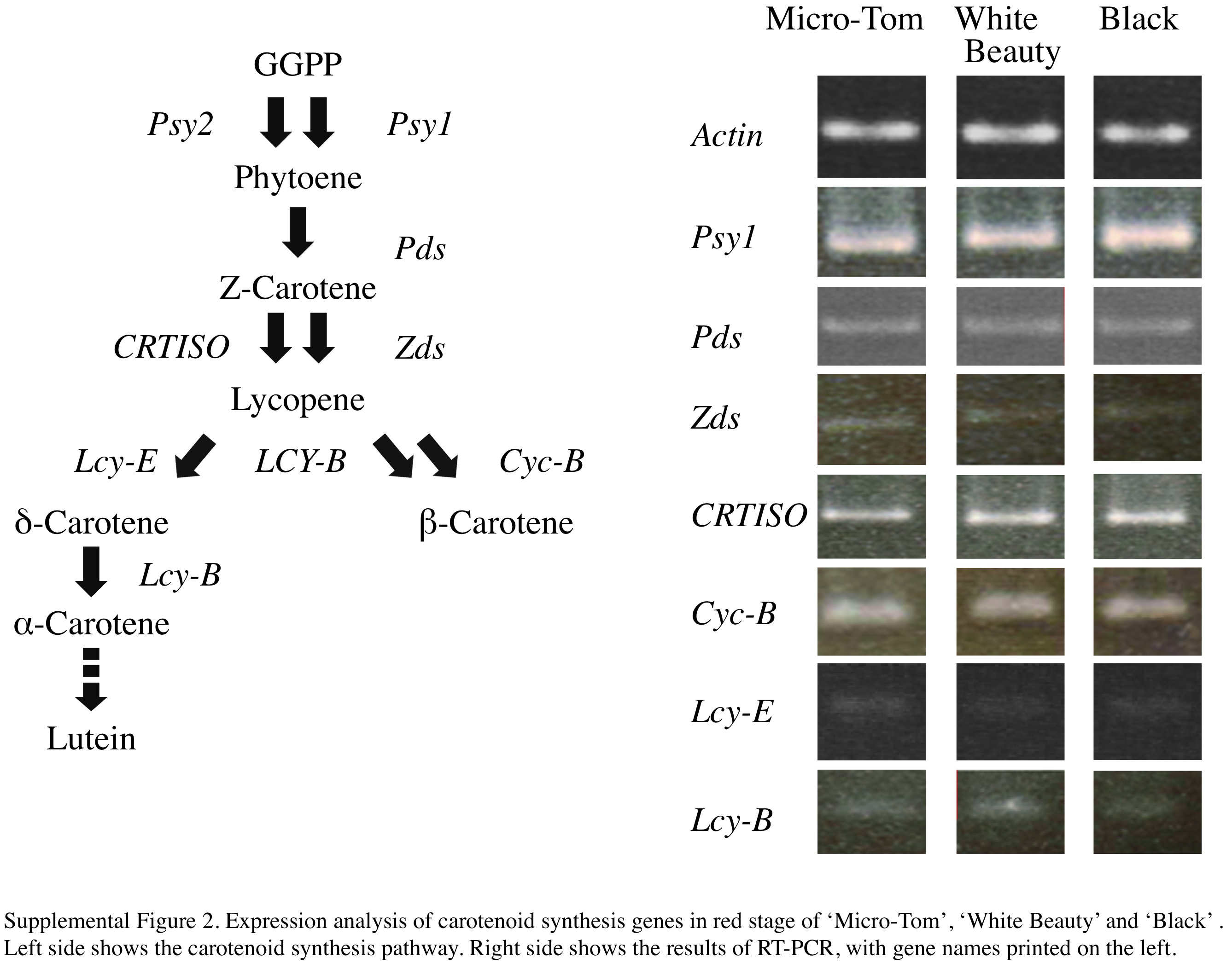

Supplement: S2 Fig — Left side shows the carotenoid synthesis pathway. Right side shows the results of RT-PCR, with gene names printed on the left. (TIF) [file pone.0137266.s005.tif]
